# Recent Advances in Understanding Cholangiocarcinoma

**DOI:** 10.12688/f1000research.12118.1

**Published:** 2017-10-09

**Authors:** Lindsey Kennedy, Laura Hargrove, Jennifer Demieville, Nicole Francis, Rowan Seils, Sara Villamaria, Heather Francis

**Affiliations:** 1 Department of Internal Medicine, Texas A&M Health Science Center, College of Medicine, Bryan, TX, USA; 2 Research, Central Texas Veterans Health Care System, Temple, TX, USA; 3 Baylor Scott & White Health Digestive Disease Research Center, Temple, TX, USA

**Keywords:** cholangiocarcinoma, microRNAs, cancer stem cells, mesenchymal stem cells

## Abstract

Cholangiocarcinoma (CCA) is an aggressive malignancy that arises from damaged epithelial cells, cholangiocytes, and possibly de-differentiated hepatocytes. CCA has a poor overall survival rate and limited therapeutic options. Based on this data, it is imperative that new diagnostic and therapeutic interventions be developed. Recent work has attempted to understand the pathological mechanisms driving CCA progression. Specifically, recent publications have delved into the role of cancer stem cells (CSCs), mesenchymal stem cells (MSCs), and microRNAs (miRNAs) during CCA pathology. CSCs are a specific subset of cells within the tumor environment that are derived from a cell with stem-like properties and have been shown to influence recurrence and chemoresistance during CCA. MSCs are known for their anti-inflammatory activity and have been postulated to influence malignancy during CCA, but little is known about their exact functions. miRNAs exert various functions via gene regulation at both the transcriptional and the translational levels, giving miRNAs diverse roles in CCA progression. Additionally, current miRNA-based therapeutic approaches are in clinical trials for various liver diseases, giving hope for similar approaches for CCA. However, the interactions among these three factors in the context of CCA are unknown. In this review, we focus on recently published data (within the last 3 years) that discuss the role of CSCs, MSCs, and miRNAs and their possible interactions during CCA pathogenesis.

## Introduction

**Figure 1.**
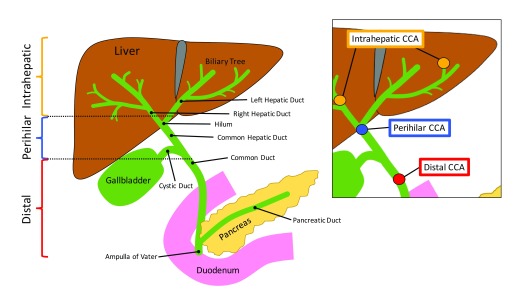
Schematic image of the anatomical locations of intrahepatic, perihilar, and distal cholangiocarcinoma (CCA). CCA is categorized on the basis of its location within the biliary tree. Intrahepatic CCA is found proximal to the second-order bile ducts, perihilar CCA is found between the second-order bile ducts and the convergence of the cystic duct and the common duct, and distal CCA is found between the cystic duct and ampulla of Vater.

Cholangiocarcinoma (CCA) is a hepatobiliary malignancy with a poor 5-year survival rate and limited treatment options. CCA arises from damaged cholangiocytes, the epithelial cells that line the biliary tree. CCA can be classified into intrahepatic, perihilar, or distal subtypes ^1^. Intrahepatic CCA, the second most common form of liver cancer, is generally located proximal to the second-order bile ducts, perihilar CCA is located between the second-order bile ducts and the intersection of the cystic duct into the common bile duct, and distal CCA is found in areas between the cystic duct and the ampulla of Vater ( Figure 1) ^2^. Aside from being categorized by anatomic location, CCA is categorized on the basis of histopathological analysis and by growth-type patterns as well ^3, 4^. For instance, CCA that has been derived from large cholangiocytes is predominantly categorized as perihilar CCA with well to moderately differentiated mucin-producing cells and periductal infiltrating growth pattern, whereas CCA that is derived from small cholangiocytes is predominantly categorized as intrahepatic CCA and largely contains non–mucin-producing cells and has mass-forming growth patterns ^3, 4^. CCA is highly heterogeneous not only in initiation and location but also in progression, making it difficult to categorize CCA into distinct subtypes.

CCA incidence has been steadily increasing ^5^. One of the first lines of treatment for CCA is resection; however, options for resection are limited since the disease is generally too advanced at the time of diagnosis ^6^. Recently, it has been reported that following resection in patients with intrahepatic CCA the 5-year and 10-year survival rates are 32.3% and 8.4%, respectively ^7^. If unresectable, liver transplantation is another option for patients with intrahepatic CCA, and post-liver transplantation 5-year survival rates are 51% ^8^. For patients with perihilar CCA, resection may be an option but outcomes are poor; 5-year survival rates are 10% ^9^. It has also been suggested that some subsets of perihilar CCA may benefit from neoadjuvant chemoradiation therapy or biliary drainage or both, but outcomes are variable ^10– 12^. For patients with distal CCA following resection, 5-year survival rates are 23% ^9^. Furthermore, these patients should not be subjected to biliary drainage, since they tend to have increased complications related to this surgery ^13^. It is apparent that our current approaches for CCA are lacking, as demonstrated by the poor survival rates and limited treatment options. For these reasons, it is critical that new and effective therapies be developed.

The CCA microenvironment contains a plethora of cell types, extracellular structures, and secreted factors that influence the progression of the tumor. Cancer cells are surrounded by stroma, which is made up of various stromal cells and extracellular matrix ^14^. Additionally, cytokines, chemokines, growth factors, and proteinases secreted from cancer cells and stromal cells can promote a pro-inflammatory environment ^14^. This type of tumor microenvironment can lead to myofibroblast activation, cancer stem cell (CSC) initiation, and the recruitment of various inflammatory cell types ^14^. The CCA microenvironment is a complex network that is influenced by a multitude of soluble factors and cell types.

This review will focus on recent publications (within the last 3 years) that advance our understanding of CCA and will analyze how their findings could lead to new diagnostic or therapeutic targets. Overall, these publications discuss their findings within the context of all three subtypes of CCA (intrahepatic, perihilar, and distal), and for this reason we discuss the findings of the articles in the context of general CCA. Among the recent publications, many of the articles fell into similar categories. Specifically, publications tended to focus on the functional and mechanistic roles of (i) CSCs, (ii) mesenchymal stem cells (MSCs), or (iii) microRNAs (miRNAs) in CCA progression. We will be discussing how these three categories influence the CCA microenvironment, either alone or in relation to one another.

## Cancer stem cells

The tumor microenvironment comprises many different cell types with various functions. Recently, CSCs have been identified in the tumor microenvironment and add complexity to our understanding of CCA. Specifically, CSCs have been identified for their role in tumor initiation, progression, and relapse. CSCs function in a manner similar to normal stem cells, wherein they are capable of self-renewal, can differentiate into multiple different cell types, and have unlimited division ^15^. CSCs promote an inflammatory environment through the activation of stromal cells and the recruitment of inflammatory cells ^16^. The cell type from which CSCs are derived is still under debate, but it has been hypothesized that CSCs may be derived from a stem-like cell that acquires a cancer-promoting alteration ^17^. Multiple studies have found that hepatocellular carcinoma (HCC) progression is driven by CSCs ^18– 21^; however, little is known about the role of CSCs in CCA.

As previously stated, the CCA microenvironment is a complex network of various cell types. Among the various cell types, macrophages are associated with CCA tumor progression and are significantly correlated with a poorer prognosis and metastasis ^4^. A recent article by Raggi *et al*. hypothesized that CSCs secrete factors that promote macrophage differentiation toward the tumor-associated macrophage (TAM) subtype ^22^. First, CCA spheres were cultured and medium was collected and placed on cultured macrophages. Following treatment with CCA sphere medium, macrophages expressed cluster of differentiation 68 (CD68), CD115, human leukocyte antigen-D related, and CD206, indicating an activated phenotype. These activated macrophages had TAM-like features, including increased invasiveness. To identify whether these findings were clinically relevant, macrophages were isolated from human CCA resections, and it was noted that these isolated macrophages expressed a phenotype similar to that of the treated cultured macrophages. The authors then identified the factors secreted from CCA spheres that were driving this TAM-like phenotype and found that interleukin-13 (IL-13), IL-34, and osteoactivin were detected in CCA sphere medium and in serum of patients with CCA. Furthermore, these factors were associated with CSC presence, and the authors concluded that the secreted factors driving the TAM-like phenotype must be derived from CSCs. These findings present a novel mechanism by which CCA-associated CSCs influence the activation of TAMs that promote CCA progression ^22^. Given that much of the work was done with CCA sphere medium, more work regarding the exact factors secreted from CSCs is necessary to definitively demonstrate that the activation of TAMs is specifically driven by CSCs.

Laminins are a family of extracellular matrix proteins that are mainly found in the basement membrane and are composed of α, β, and γ chains ^23, 24^. The γ2-chain of laminin-332 (Ln-332) is highly elevated in HCCs expressing the biliary marker cytokeratin-19 (CK-19) and is correlated with a poor prognosis for patients with HCC or intrahepatic CCA ^25– 27^. The aim of the publication by Govaere *et al*. was to characterize the CSC niche and determine the possible cell of origin ^28^. This publication slightly deviates from the others discussed in this review because it looks at CSCs in mixed HCC/CCA samples. It was noted that elevated biliary and hepatic progenitor cell (HPC) markers were seen with increased expression of *LAMC2*, the gene that encodes the γ2-chain of Ln-332. Immunopositivity for the γ2-chain of Ln-332 was found surrounding small HPC-like tumor cells that had a low proliferation rate, identified as possible CSCs. Lastly, the γ2-chain of Ln-332 was strongly co-expressed in ductular areas with low proliferative capacity but was expressed at low levels in the hepatocyte areas of HCC/CCA. The authors concluded that Ln-332 maintains the CSC niche and supports stem-like properties in these cells ^28^. While this article is one of a few that looks at the factors that maintain CSCs, future work needs to be performed to look at the role of Ln-332 specifically in CCA samples.

Given these findings, it is evident that the formation of CSCs strongly impacts the surrounding tumor microenvironment, which can impact disease progression. Specifically, these articles identify the pro-inflammatory role of CSCs during CCA. First, CSCs were shown to promote macrophage transition to a TAM-like phenotype. Furthermore, CSC accumulation of laminins, a key component of extracellular matrix, may promote the survival, migration, and invasion of tumor cells as well as stromal activation. It is evident that CSCs are key in maintaining and promoting a pro-inflammatory environment during CCA initiation and progression.

## Mesenchymal stem cells

Recently, interest in the role of MSCs in tumor progression and metastasis has increased. MSCs are non-hematopoietic stem cells that primarily reside in the bone marrow but are recruited to injured tissues, inflammatory sites, and primary tumors ^29– 31^. As stated above, the presence of CSCs promotes an inflammatory environment, which may promote MSC migration and the infiltration of CCA tumors. MSCs have low immunogenicity and, following recruitment to injured tissues, are able to maintain their multi-differentiation capacity ^32^. Specifically, MSCs may differentiate into cancer-associated fibroblasts, myofibroblasts, or hepatic stellate cells to promote tumor progression ^33, 34^. These various cell types increase the inflammatory capacity of the stromal environment, further promoting the initiation of CSCs and the recruitment of MSCs. Moreover, MSCs secrete various cytokines that promote an inflammatory microenvironment ^35, 36^. For these reasons, the role of MSCs in CCA progression and inflammatory response has become a topic of interest.

While MSCs are known to influence the inflammatory environment of tumors, the exact microenvironment necessary for these effects is unknown. Also, it has been postulated that inflammatory conditions are a major activator of MSC-associated immunosuppression ^37^. Recent work from Zhong *et al*. found that tumor necrosis factor-alpha (TNF-α) and interferon-gamma (IFN-γ) were able to stimulate the expression of TNF-α, C-C motif chemokine ligand 5 (CCL5), IL-6 and indoleamine 2,3-dioxygenase, and activated nuclear factor kappa B (NF-κB) signaling in MSCs ^38^. Secreted factors from these stimulated MSCs were able to induce CCA cell migration and metastasis *in vitro* and *in vivo*. Furthermore, CCA cells treated with supernatants from the stimulated MSCs had increased expression of C-C motif chemokine receptor 5 (CCR5). Increased CCL5/CCR5 signaling in CCA cells was able to increase the expression of matrix metalloproteinase-2 (MMP-2) and MMP-9. Overall, the authors deduced that TNF-α and IFN-γ stimulate MSCs to secrete CCL5, which in turn activates CCR5 on CCA cells to promote inflammation and metastasis ^38^. Inhibition of TNF-α or IFN-γ signaling or both could block MSC recruitment, thereby reducing CCA inflammation and metastasis to hopefully contribute to an improved outcome.

Chemoresistance is a major obstacle in cancer treatment. Previously, it was demonstrated that MSC-secreted IL-8 induces doxorubicin resistance in triple-negative breast cancer ^39^. Considering these findings, Wang *et al*. examined the role of MSCs in the progression of CCA development ^40^. Using human umbilical cord–derived MSCs, the authors noted that cultured CCA cells that were co-cultured with these MSCs had increased cell proliferation, metastasis, and resistance to the anti-cancer drug compound K *in vivo*. *In vitro*, CCA cells co-cultured with MSCs had increased colony formation and invasion. The tumors from mice injected with CCA cells co-cultured with MSCs demonstrated enhanced Wnt/β-catenin signaling. These findings were corroborated *in vitro* where MSCs and their secreted factors were shown to stimulate Wnt signaling via nuclear translocation of β-catenin, upregulation of Wnt, and increased expression of the downstream targets MMP-2, cyclin D1, and c-Myc in cultured CCA cells. Overall, this study indicates that MSCs increase CCA metastasis and chemoresistance via increased Wnt/β-catenin signaling ^40^. Targeting the Wnt/β-catenin signaling pathway may prove therapeutic for patients with CCA.

It is interesting to note that TNF-α and IFN-γ are necessary to drive MSC migration to CCA tumors. Given that CSCs promote an inflammatory environment, this may play a role in the recruitment of MSCs to the CCA tumor. Based on these articles, the presence of MSCs in CCA is indicative of increased CCA migration and invasion, and inhibition of MSC migration or induction of MSC apoptosis may be therapeutic for patients with CCA.

## microRNAs

miRNAs are short, non-coding RNAs that regulate the expression of specific mRNAs ^41^. Specifically, miRNAs will recognize and bind to complementary sequences found within the 3′ untranslated region (3′ UTR) of specific mRNAs to regulate their expression levels ^42, 43^. The role of miRNAs varies, depending on the cellular process, in both physiological and pathological conditions ^44– 46^. The function of miRNAs during CCA has increasingly become a topic of interest; multiple human clinical trials evaluating the efficacy of miRNA-based therapeutics during various liver diseases are under way ^47, 48^. miRNA signaling has been noted in all three subtypes of CCA ^49– 51^; however, the identification of subtype-specific miRNAs has not been made. It is known that miRNAs can regulate MSC and CSC function ^52– 54^, but this regulation has not been reported in the context of CCA. Further understanding the impact of miRNAs on CCA development and progression may bring about novel diagnostic tools or therapeutic interventions. In this section, we will be discussing the role of miRNAs during CCA progression but also analyzing their potential impacts on CSC and MSC biology.

Menin is a known tumor-suppressor gene that is expressed in all tissues ^55^, but its role in CCA is poorly defined. miR-24 has previously been recognized as an oncogene in multiple gastrointestinal cancers and has been shown to target menin, but this interaction in the context of CCA is not understood ^56– 59^. A recent article by Ehrlich *et al*. evaluated the role of menin during CCA proliferation and angiogenesis and its regulation by miR-24 ^51^. The authors found that human advanced CCA tumor sections, as well as *in vitro* human CCA cell lines, had increased miR-24 expression alongside decreased menin expression. *In vitro*, human CCA cell lines treated with an miR-24 inhibitor showed increased menin levels with a subsequent reduction in cell proliferation, angiogenesis, migration, and invasion. It was then demonstrated that miR-24 negatively regulates menin expression. *In vivo*, the authors noted that tumor size and the expression of proliferative and angiogenic markers were significantly reduced in the miR-24–inhibited tumor group compared with controls. Interestingly, fibrogenesis was enhanced in the miR-24–inhibited tumor group when compared with controls. From this study, it is evident that miR-24 acts as an oncogene to suppress menin expression, thereby increasing tumor burden, proliferation, angiogenesis, migration, and invasion ^51^. However, further work is necessary to delineate the exact downstream mechanisms regulating these events as well as understand the primary cellular source of miR-24 during CCA, which may help us understand why fibrogenesis increased even as tumor burden and angiogenesis decreased. While this study focuses on the role of miR-24 in cultured CCA cells, miR-24 has previously been shown to promote stemness in embryonic stem cells ^60^. In this context, miR-24 may promote the formation of CSCs in CCA, leading to increased CCA migration and invasion.

Circadian rhythms are endogenous oscillations that are regulated by clock genes and are present in the central nervous system, peripheral tissues, and single cells ^61^. While circadian oscillations are a normal physiological process, dysregulation of these oscillations promotes tumor development ^62, 63^; however, little is known about the role of the circadian rhythm during CCA. A recent publication identified that the expression of Per1, which negatively regulates circadian oscillations, was decreased in human CCA samples and cultured human CCA cell lines ^64^. In addition, overexpression of Per1 decreased cell proliferation and increased apoptosis in cultured human CCA cells. These findings were mimicked *in vivo*, wherein immunocompromised mice injected with cultured human CCA cells overexpressing Per1 had decreased tumor growth, proliferation, angiogenesis, and metastasis. Per1 was found to be a target of miR-34a, and following treatment with a miR-34a inhibitor, human cultured CCA cells had decreased proliferation, migration, and invasion. These findings are the first to identify that the disruption of clock genes regulated by miR-34a contributes to CCA malignancy ^64^. As stated, circadian clock genes are present in all cells and tissues, and previous work has shown that circadian clock genes regulate MSC differentiation, migration, and cell cycle ^65^. It is possible that the regulation of core clock genes via miR-34a plays a role in MSC migration and activation during CCA. Also, the disruption of core clock genes in CCA cells may contribute to the tumor niche, thereby supporting CSC formation or MSC recruitment or both.

Tumor growth and metastatic potential are regulated by a myriad of factors, including epigenetic changes, such as DNA methylation ^66, 67^. Specifically, it has been shown that DNA methylation plays a prominent role in the progression of CCA ^68^. On the basis of this information, Zhou *et al*. set out to investigate the functional and mechanistic role of miR-191 in CCA ^69^. It was first noted that miR-191 expression was increased in CCA tumors when compared with adjacent normal bile duct tissue. miR-191 expression was found to be an independent risk factor for a worse prognosis for human CCA patients. *In vivo* and *in vitro* analysis revealed that overexpression of miR-191 was associated with enhanced proliferation, invasion, and migration and reduced ten-eleven translocation 1 (TET1) expression, which induces DNA demethylation and was shown to be a direct target of miR-191. The authors then found that TET1 expression allows for the methylation of CpG-rich regions in the gene transcription start site of p53, a tumor suppressor, leading to a reduction of p53 expression and subsequent increase in tumor burden. These findings suggest that the overexpression of miR-191 is associated with CCA progression via miR-191/TET1/p53 signaling ^69^. Aside from its role in p53 expression ^70^, miR-191 has been shown to promote CSC-like properties in bronchial epithelial cells, alluding to the fact that miR-191 may influence the CSC niche in CCA as well. This publication identifies a novel therapeutic target for the treatment of CCA and suggests that miR-191 levels may be used as a diagnostic or prognostic factor or both.

Aside from p53, N-myc downstream-regulated gene 2 (NDRG2) is another tumor suppressor that plays a role in the progression of various cancers ^71, 72^. NDRG2 has been shown to be downregulated during tumor progression, and it has been postulated that NDRG2 inhibits the metastasis of HCC ^71– 73^. miR-181 is upregulated in several cancers ^74– 76^; however, miR-181 has also been shown to inhibit tumor formation ^77– 79^. Leukemia inhibitory factor (LIF) is a member of the IL-6 cytokine family whose dysregulation has been observed in different cancers ^79^. The potential function of NDRG2, LIF, and miR-181c in the development and progression of CCA is not fully understood, and a recent publication evaluated the potential roles and mechanisms of these factors ^80^. The authors found that the expression of NDRG2 was decreased and miR-181c and LIF increased in human CCA compared with non-tumor tissues. Furthermore, downregulation of NDRG2 alongside overexpression of miR-181c or LIF indicated a poorer overall survival in patients with CCA. *In vivo* and *in vitro*, it was shown that overexpression of NDRG2 was able to inhibit CCA cell proliferation, chemoresistance, and metastasis. LIF was able to activate miR-181c, while NDRG2 inhibited LIF transcription. These findings identify that NDRG2 and LIF/miR-181c counteract each other, and dysregulation of one of these pathways may contribute to carcinogenesis and metastasis ^80^. Given that LIF is an IL-6 cytokine, miR-181c regulation of LIF may influence the inflammatory properties of the microenvironment so that it favors CSC formation or MSC recruitment. Though complex, this novel pathway may serve as a potential therapeutic target for the treatment of CCA.

The role of miR-16 in tumorigenesis has been studied in various cancers ^81– 83^, but its role in CCA is unknown. Yes-associated protein 1 (YAP1) is inhibited by the hippo signaling pathway, but in the absence of this inhibition, YAP1 acts as an oncogene in numerous cancers ^84– 88^; however, mechanisms regulating YAP1 expression in human CCA are largely unknown. Recent work found that in human CCA tissues miR-16 expression was significantly downregulated and correlated with tumor size, metastasis, and staging ^89^. Furthermore, downregulation of miR-16 was strongly associated with increased tumor progression and worsened overall survival in human CCA patients. *In vivo* and *in vitro* experimentation demonstrated that overexpression of miR-16 inhibited CCA cell proliferation, invasion, and metastasis. The authors then found that YAP1 was a direct target of miR-16. In support of these findings, it was noted that YAP1 levels are greatly enhanced in human CCA tissues, which was inversely correlated with the above findings that miR-16 is largely downregulated in human CCA. This publication concludes that miR-16 acts as a tumor suppressor in CCA through downregulation of YAP1 ^89^. The miR-16/YAP1 interaction may not only be a therapeutic target but also act as a reliable prognostic marker for CCA. Additionally, YAP1 has been shown to control the self-renewal and differentiation of MSCs; therefore, miR-16 regulation of YAP1 may influence MSC function during CCA ^90^.

It has long been known that miRNAs play a role in the initiation and progression of CCA, but miRNA regulation of MSCs and CSCs during CCA is unknown. While these publications discuss the role of miRNAs in CCA cells and tissues, we can draw comparisons to the impact that they may have on MSC and CSC function. Furthermore, we can note that miRNA signaling may manipulate the microenvironment to become pro-inflammatory, which will favor the initiation of CSC formation as well as the recruitment of MSCs to the injured tissue. However, the discussion of miRNA regulation of MSCs and CSCs during CCA is largely speculative, and further research is necessary to fully understand this signaling process.

## Conclusions

Currently, therapeutic options for patients with CCA are extremely limited, a worrying predicament given the increase in incidence. In addition, options for patients with CCA are further limited since the disease tends to be at an advanced stage at the time of diagnosis. These facts indicate the imperative need to develop better diagnostic tools to help identify CCA at an earlier time point as well as more sophisticated therapeutic tools to improve survival rates. The roles of MSCs and CSCs during CCA are a new area of study with little information known about these cells. For this reason, more studies are needed to fully elucidate the impact that MSCs and CSCs have on CCA development and progression. It is possible that therapies targeting these cells will reduce chemoresistance and recurrence, but only future studies will be able to fully define this. There are a large number of publications regarding miRNA regulation of CCA progression; however, miRNA regulation of MSCs and CSCs during CCA is unknown. Given that miRNA therapies for various liver diseases are currently in clinical trials, there is hope that new miRNA-based therapies are developed for CCA, specifically those that may impact MSC and CSC function. While we have made strides in terms of understanding the CCA tumor environment and the pathways regulating progression, few translational findings have been developed. In the future, translational studies are necessary to help find diagnostic, prognostic, and therapeutic tools for the treatment of CCA.

## Abbreviations

CCA, cholangiocarcinoma; CCL5, C-C motif chemokine ligand 5; CCR5, C-C motif chemokine receptor 5; CD, cluster of differentiation; CSC, cancer stem cell; HCC, hepatocellular carcinoma; HPC, hepatic progenitor cell; IFN-γ, interferon-gamma; IL, interleukin; LIF, leukemia inhibitory factor; Ln-332, laminin-332; miRNA, microRNA; MMP, matrix metalloproteinase; MSC, mesenchymal stem cell; NDRG2, N-myc downstream-regulated gene 2; TAM, tumor-associated macrophage; TET1, ten-eleven translocation 1; TNF-α, tumor necrosis factor-alpha; YAP1, yes-associated protein 1.

## References

[1] Oliveira IS Kilcoyne A Everett JM: Cholangiocarcinoma: classification, diagnosis, staging, imaging features, and management. *Abdom Radiol (NY).* 2017;42(6):1637–49. 10.1007/s00261-017-1094-7 28271275

[2] Razumilava N Gores GJ: Combination of gemcitabine and cisplatin for biliary tract cancer: a platform to build on. *J Hepatol.* 2011;54(3):577–8. 10.1016/j.jhep.2010.10.010 21112109

[3] Cardinale V Bragazzi MC Carpino G: Cholangiocarcinoma: increasing burden of classifications. *Hepatobiliary Surg Nutr.* 2013;2(5):272–80. 10.3978/j.issn.2304-3881.2013.10.02 24570958 PMC3924690

[4] Banales JM Cardinale V Carpino G: Expert consensus document: Cholangiocarcinoma: current knowledge and future perspectives consensus statement from the European Network for the Study of Cholangiocarcinoma (ENS-CCA). *Nat Rev Gastroenterol Hepatol.* 2016;13(5):261–80. 10.1038/nrgastro.2016.51 27095655

[5] Bridgewater J Galle PR Khan SA: Guidelines for the diagnosis and management of intrahepatic cholangiocarcinoma. *J Hepatol.* 2014;60(6):1268–89. 10.1016/j.jhep.2014.01.021 24681130

[6] Guglielmi A Ruzzenente A Campagnaro T: Intrahepatic cholangiocarcinoma: prognostic factors after surgical resection. *World J Surg.* 2009;33(6):1247–54. 10.1007/s00268-009-9970-0 19294467

[7] Si A Li J Xiang H: Actual over 10-year survival after liver resection for patients with intrahepatic cholangiocarcinoma. *Oncotarget.* 2017;8(27):44521–32. 10.18632/oncotarget.17815 28562348 PMC5546499

[8] Elshamy M Presser N Hammad AY: Liver transplantation in patients with incidental hepatocellular carcinoma/cholangiocarcinoma and intrahepatic cholangiocarcinoma: a single-center experience. *Hepatobiliary Pancreat Dis Int.* 2017;16(3):264–70. 10.1016/S1499-3872(17)60016-X 28603094

[9] DeOliveira ML Cunningham SC Cameron JL: Cholangiocarcinoma: thirty-one-year experience with 564 patients at a single institution. *Ann Surg.* 2007;245(5):755–62. 10.1097/01.sla.0000251366.62632.d3 17457168 PMC1877058

[10] Darwish Murad S Kim WR Harnois DM: Efficacy of neoadjuvant chemoradiation, followed by liver transplantation, for perihilar cholangiocarcinoma at 12 US centers. *Gastroenterology.* 2012;143(1):88–98.e3; quiz e14. 10.1053/j.gastro.2012.04.008 22504095 PMC3846443

[11] Deviere J Baize M de Toeuf J: Long-term follow-up of patients with hilar malignant stricture treated by endoscopic internal biliary drainage. *Gastrointest Endosc.* 1988;34(2):95–101. 10.1016/S0016-5107(88)71271-7 2835282

[12] Bhat M Hathcock M Kremers WK: Portal vein encasement predicts neoadjuvant therapy response in liver transplantation for perihilar cholangiocarcinoma protocol. *Transpl Int.* 2015;28(12):1383–91. 10.1111/tri.12640 26183487

[13] van der Gaag NA Rauws EA van Eijck CH: Preoperative biliary drainage for cancer of the head of the pancreas. *N Engl J Med.* 2010;362(2):129–37. 10.1056/NEJMoa0903230 20071702

[14] Brivio S Cadamuro M Strazzabosco M: Tumor reactive stroma in cholangiocarcinoma: The fuel behind cancer aggressiveness. *World J Hepatol.* 2017;9(9):455–68. 10.4254/wjh.v9.i9.455 28396716 PMC5368623

[15] Pattabiraman DR Weinberg RA: Tackling the cancer stem cells - what challenges do they pose? *Nat Rev Drug Discov.* 2014;13(7):497–512. 10.1038/nrd4253 24981363 PMC4234172

[16] Romano M De Francesco F Gringeri E: Tumor Microenvironment Versus Cancer Stem Cells in Cholangiocarcinoma: Synergistic Effects? *J Cell Physiol.* 2016;231(4):768–76. 10.1002/jcp.25190 26357947

[17] Raggi C Invernizzi P Andersen JB: Impact of microenvironment and stem-like plasticity in cholangiocarcinoma: molecular networks and biological concepts. *J Hepatol.* 2015;62(1):198–207. 10.1016/j.jhep.2014.09.007 25220250

[18] Marquardt JU Raggi C Andersen JB: Human hepatic cancer stem cells are characterized by common stemness traits and diverse oncogenic pathways. *Hepatology.* 2011;54(3):1031–42. 10.1002/hep.24454 21618577 PMC3179780

[19] Ma S Chan KW Hu L: Identification and characterization of tumorigenic liver cancer stem/progenitor cells. *Gastroenterology.* 2007;132(7):2542–56. 10.1053/j.gastro.2007.04.025 17570225

[20] Raggi C Factor VM Seo D: Epigenetic reprogramming modulates malignant properties of human liver cancer. *Hepatology.* 2014;59(6):2251–62. 10.1002/hep.27026 24449497 PMC4043911

[21] Yamashita T Forgues M Wang W: EpCAM and alpha-fetoprotein expression defines novel prognostic subtypes of hepatocellular carcinoma. *Cancer Res.* 2008;68(5):1451–61. 10.1158/0008-5472.CAN-07-6013 18316609

[22] Raggi C Correnti M Sica A: Cholangiocarcinoma stem-like subset shapes tumor-initiating niche by educating associated macrophages. *J Hepatol.* 2017;66(1):102–15. 10.1016/j.jhep.2016.08.012 27593106 PMC5522599

[23] Kallis YN Robson AJ Fallowfield JA: Remodelling of extracellular matrix is a requirement for the hepatic progenitor cell response. *Gut.* 2011;60(4):525–33. 10.1136/gut.2010.224436 21106552

[24] Lorenzini S Bird TG Boulter L: Characterisation of a stereotypical cellular and extracellular adult liver progenitor cell niche in rodents and diseased human liver. *Gut.* 2010;59(5):645–54. 10.1136/gut.2009.182345 20427399 PMC3034133

[25] Govaere O Komuta M Berkers J: Keratin 19: a key role player in the invasion of human hepatocellular carcinomas. *Gut.* 2014;63(4):674–85. 10.1136/gutjnl-2012-304351 23958557 PMC3963546

[26] Sulpice L Rayar M Desille M: Molecular profiling of stroma identifies osteopontin as an independent predictor of poor prognosis in intrahepatic cholangiocarcinoma. *Hepatology.* 2013;58(6):1992–2000. 10.1002/hep.26577 23775819

[27] Giannelli G Fransvea E Bergamini C: Laminin-5 chains are expressed differentially in metastatic and nonmetastatic hepatocellular carcinoma. *Clin Cancer Res.* 2003;9(10 Pt 1):3684–91. 14506159

[28] Govaere O Wouters J Petz M: Laminin-332 sustains chemoresistance and quiescence as part of the human hepatic cancer stem cell niche. *J Hepatol.* 2016;64(3):609–17. 10.1016/j.jhep.2015.11.011 26592953

[29] Karp JM Leng Teo GS: Mesenchymal stem cell homing: the devil is in the details. *Cell Stem Cell.* 2009;4(3):206–16. 10.1016/j.stem.2009.02.001 19265660

[30] Chamberlain G Fox J Ashton B: Concise review: mesenchymal stem cells: their phenotype, differentiation capacity, immunological features, and potential for homing. *Stem Cells.* 2007;25(11):2739–49. 10.1634/stemcells.2007-0197 17656645

[31] Spaeth E Klopp A Dembinski J: Inflammation and tumor microenvironments: defining the migratory itinerary of mesenchymal stem cells. *Gene Ther.* 2008;15(10):730–8. 10.1038/gt.2008.39 18401438

[32] Yagi H Soto-Gutierrez A Parekkadan B: Mesenchymal stem cells: Mechanisms of immunomodulation and homing. *Cell Transplant.* 2010;19(6):667–79. 10.3727/096368910X508762 20525442 PMC2957533

[33] Spaeth EL Dembinski JL Sasser AK: Mesenchymal stem cell transition to tumor-associated fibroblasts contributes to fibrovascular network expansion and tumor progression. *PLoS One.* 2009;4(4):e4992. 10.1371/journal.pone.0004992 19352430 PMC2661372

[34] Sawitza I Kordes C Götze S: Bile acids induce hepatic differentiation of mesenchymal stem cells. *Sci Rep.* 2015;5:13320. 10.1038/srep13320 26304833 PMC4548444

[35] Keating A: Mesenchymal stromal cells: new directions. *Cell Stem Cell.* 2012;10(6):709–16. 10.1016/j.stem.2012.05.015 22704511

[36] Nauta AJ Fibbe WE: Immunomodulatory properties of mesenchymal stromal cells. *Blood.* 2007;110(10):3499–506. 10.1182/blood-2007-02-069716 17664353

[37] Krampera M Cosmi L Angeli R: Role for interferon-gamma in the immunomodulatory activity of human bone marrow mesenchymal stem cells. *Stem Cells.* 2006;24(2):386–98. 10.1634/stemcells.2005-0008 16123384

[38] Zhong W Tong Y Li Y: Mesenchymal stem cells in inflammatory microenvironment potently promote metastatic growth of cholangiocarcinoma via activating Akt/NF-κ B signaling by paracrine CCL5. *Oncotarget.* 2017;8:73693–73704. 10.18632/oncotarget.17793 PMC5650292 29088737

[39] Chen DR Lu DY Lin HY: Mesenchymal stem cell-induced doxorubicin resistance in triple negative breast cancer. *Biomed Res Int.* 2014;2014: 532161. 10.1155/2014/532161 25140317 PMC4124237

[40] Wang W Zhong W Yuan J: Involvement of Wnt/β-catenin signaling in the mesenchymal stem cells promote metastatic growth and chemoresistance of cholangiocarcinoma. *Oncotarget.* 2015;6(39):42276–89. 10.18632/oncotarget.5514 26474277 PMC4747224

[41] Friedman RC Farh KK Burge CB: Most mammalian mRNAs are conserved targets of microRNAs. *Genome Res.* 2009;19(1):92–105. 10.1101/gr.082701.108 18955434 PMC2612969

[42] Pillai RS Artus CG Filipowicz W: Tethering of human Ago proteins to mRNA mimics the miRNA-mediated repression of protein synthesis. *RNA.* 2004;10(10):1518–25. 10.1261/rna.7131604 15337849 PMC1370638

[43] Bartel DP: MicroRNAs: target recognition and regulatory functions. *Cell.* 2009;136(2):215–33. 10.1016/j.cell.2009.01.002 19167326 PMC3794896

[44] Katsumi T Ninomiya M Nishina T: MiR-139-5p is associated with inflammatory regulation through c-FOS suppression, and contributes to the progression of primary biliary cholangitis. *Lab Invest.* 2016;96(11):1165–77. 10.1038/labinvest.2016.95 27668889

[45] Francis H McDaniel K Han Y: Regulation of the extrinsic apoptotic pathway by microRNA-21 in alcoholic liver injury. *J Biol Chem.* 2014;289(40):27526–39. 10.1074/jbc.M114.602383 25118289 PMC4183793

[46] Kennedy LL Meng F Venter JK: Knockout of microRNA-21 reduces biliary hyperplasia and liver fibrosis in cholestatic bile duct ligated mice. *Lab Invest.* 2016;96(12):1256–67. 10.1038/labinvest.2016.112 27775690 PMC5121007

[47] Shibata C Otsuka M Kishikawa T: Current status of miRNA-targeting therapeutics and preclinical studies against gastroenterological carcinoma. *Mol Cell Ther.* 2013;1:5. 10.1186/2052-8426-1-5 26056570 PMC4448951

[48] Baek J Kang S Min H: MicroRNA-targeting therapeutics for hepatitis C. *Arch Pharm Res.* 2014;37(3):299–305. 10.1007/s12272-013-0318-9 24385319

[49] Palumbo T Poultsides GA Kouraklis G: A functional microRNA library screen reveals miR-410 as a novel anti-apoptotic regulator of cholangiocarcinoma. *BMC Cancer.* 2016;16(1):353. 10.1186/s12885-016-2384-0 27259577 PMC4893280

[50] Haga H Yan I Takahashi K: Emerging insights into the role of microRNAs in the pathogenesis of cholangiocarcinoma. *Gene Expr.* 2014;16(2):93–9. 10.3727/105221614X13919976902174 24801170 PMC4166576

[51] Ehrlich L Hall C Venter J: miR-24 Inhibition Increases Menin Expression and Decreases Cholangiocarcinoma Proliferation. *Am J Pathol.* 2017;187(3):570–80. 10.1016/j.ajpath.2016.10.021 28087162 PMC5389363

[52] Du K Li Z Fang X: Ferulic acid promotes osteogenesis of bone marrow-derived mesenchymal stem cells by inhibiting microRNA-340 to induce β-catenin expression through hypoxia. *Eur J Cell Biol.* 2017;96(6):496–503. 10.1016/j.ejcb.2017.07.002 28764862

[53] Pakravan K Babashah S Sadeghizadeh M: MicroRNA-100 shuttled by mesenchymal stem cell-derived exosomes suppresses *in vitro* angiogenesis through modulating the mTOR/HIF-1α/VEGF signaling axis in breast cancer cells. *Cell Oncol (Dordr).* 2017;40(5):457–470. 10.1007/s13402-017-0335-7 28741069 PMC13001539

[54] Yu M Xue Y Zheng J: Linc00152 promotes malignant progression of glioma stem cells by regulating miR-103a-3p/FEZF1/CDC25A pathway. *Mol Cancer.* 2017;16(1):110. 10.1186/s12943-017-0677-9 28651608 PMC5485714

[55] Chandrasekharappa SC Guru SC Manickam P: Positional cloning of the gene for multiple endocrine neoplasia-type 1. *Science.* 1997;276(5311):404–7. 10.1126/science.276.5311.404 9103196

[56] Vijayaraghavan J Maggi EC Crabtree JS: miR-24 regulates menin in the endocrine pancreas. *Am J Physiol Endocrinol Metab.* 2014;307(1):E84–92. 10.1152/ajpendo.00542.2013 24824656

[57] Meng FL Wang W Jia WD: Diagnostic and prognostic significance of serum miR-24-3p in HBV-related hepatocellular carcinoma. *Med Oncol.* 2014;31(9):177. 10.1007/s12032-014-0177-3 25129312

[58] Liu YX Long XD Xi ZF: MicroRNA-24 modulates aflatoxin B1-related hepatocellular carcinoma prognosis and tumorigenesis. *Biomed Res Int.* 2014;2014:482926. 10.1155/2014/482926 24800232 PMC3997078

[59] Dong W Li B Wang Z: Clinical significance of microRNA-24 expression in esophageal squamous cell carcinoma. *Neoplasma.* 2015;62(2):250–8. 10.4149/neo_2015_030 25591590

[60] Lee SH Chen TY Dhar SS: A feedback loop comprising PRMT7 and miR-24-2 interplays with Oct4, Nanog, Klf4 and c-Myc to regulate stemness. *Nucleic Acids Res.* 2016;44(22):10603–18. 10.1093/nar/gkw788 27625395 PMC5159542

[61] Balsalobre A Damiola F Schibler U: A serum shock induces circadian gene expression in mammalian tissue culture cells. *Cell.* 1998;93(6):929–37. 10.1016/S0092-8674(00)81199-X 9635423

[62] Filipski E King VM Li X: Host circadian clock as a control point in tumor progression. *J Natl Cancer Inst.* 2002;94(9):690–7. 10.1093/jnci/94.9.690 11983758

[63] Viswanathan AN Schernhammer ES: Circulating melatonin and the risk of breast and endometrial cancer in women. *Cancer Lett.* 2009;281(1):1–7. 10.1016/j.canlet.2008.11.002 19070424 PMC2735793

[64] Han Y Meng F Venter J: miR-34a-dependent overexpression of *Per1* decreases cholangiocarcinoma growth. *J Hepatol.* 2016;64(6):1295–304. 10.1016/j.jhep.2016.02.024 26923637 PMC4874896

[65] Boucher H Vanneaux V Domet T: Circadian Clock Genes Modulate Human Bone Marrow Mesenchymal Stem Cell Differentiation, Migration and Cell Cycle. *PLoS One.* 2016;11(1):e0146674. 10.1371/journal.pone.0146674 26741371 PMC4704833

[66] Dawson MA Kouzarides T: Cancer epigenetics: from mechanism to therapy. *Cell.* 2012;150(1):12–27. 10.1016/j.cell.2012.06.013 22770212

[67] You JS Jones PA: Cancer genetics and epigenetics: two sides of the same coin? *Cancer Cell.* 2012;22(1):9–20. 10.1016/j.ccr.2012.06.008 22789535 PMC3396881

[68] Sandhu DS Shire AM Roberts LR: Epigenetic DNA hypermethylation in cholangiocarcinoma: potential roles in pathogenesis, diagnosis and identification of treatment targets. *Liver Int.* 2008;28(1):12–27. 10.1111/j.1478-3231.2007.01624.x 18031477 PMC2904912

[69] Li H Zhou ZQ Yang ZR: MicroRNA-191 acts as a tumor promoter by modulating the TET1-p53 pathway in intrahepatic cholangiocarcinoma. *Hepatology.* 2017;66(1):136–51. 10.1002/hep.29116 28194813

[70] Xu W Ji J Xu Y: MicroRNA-191, by promoting the EMT and increasing CSC-like properties, is involved in neoplastic and metastatic properties of transformed human bronchial epithelial cells. *Mol Carcinog.* 2015;54 Suppl 1:E148–61. 10.1002/mc.22221 25252218

[71] Lee DC Kang YK Kim WH: Functional and clinical evidence for *NDRG2* as a candidate suppressor of liver cancer metastasis. *Cancer Res.* 2008;68(11):4210–20. 10.1158/0008-5472.CAN-07-5040 18519680

[72] Tepel M Roerig P Wolter M: Frequent promoter hypermethylation and transcriptional downregulation of the *NDRG2* gene at 14q11.2 in primary glioblastoma. *Int J Cancer.* 2008;123(9):2080–6. 10.1002/ijc.23705 18709645

[73] Wang J Yin D Xie C: The iron chelator Dp44mT inhibits hepatocellular carcinoma metastasis via N-Myc downstream-regulated gene 2 (NDRG2)/gp130/STAT3 pathway. *Oncotarget.* 2014;5(18):8478–91. 10.18632/oncotarget.2328 25261367 PMC4226698

[74] Tong SJ Liu J Wang X: microRNA-181 promotes prostate cancer cell proliferation by regulating DAX-1 expression. *Exp Ther Med.* 2014;8(4):1296–300. 10.3892/etm.2014.1846 25187843 PMC4151665

[75] Swanson JM Wigal S Greenhill LL: Analog classroom assessment of Adderall in children with ADHD. *J Am Acad Child Adolesc Psychiatry.* 1998;37(5):519–26. 10.1097/00004583-199805000-00014 9585654

[76] Mansueto G Forzati F Ferraro A: Identification of a New Pathway for Tumor Progression: MicroRNA-181b Up-Regulation and CBX7 Down-Regulation by HMGA1 Protein. *Genes Cancer.* 2010;1(3):210–24. 10.1177/1947601910366860 21779448 PMC3092193

[77] Huang P Ye B Yang Y: MicroRNA-181 functions as a tumor suppressor in non-small cell lung cancer (NSCLC) by targeting Bcl-2. *Tumour Biol.* 2015;36(5):3381–7. 10.1007/s13277-014-2972-z 25524579

[78] Conti A Aguennouz M La Torre D: miR-21 and 221 upregulation and miR-181b downregulation in human grade II-IV astrocytic tumors. *J Neurooncol.* 2009;93(3):325–32. 10.1007/s11060-009-9797-4 19159078

[79] Pichler M Winter E Ress AL: miR-181a is associated with poor clinical outcome in patients with colorectal cancer treated with EGFR inhibitor. *J Clin Pathol.* 2014;67(3):198–203. 10.1136/jclinpath-2013-201904 24098024

[80] Wang J Xie C Pan S: N-myc downstream-regulated gene 2 inhibits human cholangiocarcinoma progression and is regulated by leukemia inhibitory factor/MicroRNA-181c negative feedback pathway. *Hepatology.* 2016;64(5):1606–22. 10.1002/hep.28781 27533020

[81] Chatterjee A Chattopadhyay D Chakrabarti G: MiR-16 targets Bcl-2 in paclitaxel-resistant lung cancer cells and overexpression of miR-16 along with miR-17 causes unprecedented sensitivity by simultaneously modulating autophagy and apoptosis. *Cell Signal.* 2015;27(2):189–203. 10.1016/j.cellsig.2014.11.023 25435430

[82] Tang X Jin L Cao P: MicroRNA-16 sensitizes breast cancer cells to paclitaxel through suppression of IKBKB expression. *Oncotarget.* 2016;7(17):23668–83. 10.18632/oncotarget.8056 26993770 PMC5029655

[83] Chen L Wang Q Wang GD: miR-16 inhibits cell proliferation by targeting IGF1R and the Raf1-MEK1/2-ERK1/2 pathway in osteosarcoma. *FEBS Lett.* 2013;587(9):1366–72. 10.1016/j.febslet.2013.03.007 23507142

[84] Perra A Kowalik MA Ghiso E: YAP activation is an early event and a potential therapeutic target in liver cancer development. *J Hepatol.* 2014;61(5):1088–96. 10.1016/j.jhep.2014.06.033 25010260

[85] Lee K Lee SS Kim SB: Significant association of oncogene YAP1 with poor prognosis and cetuximab resistance in colorectal cancer patients. *Clin Cancer Res.* 2015;21(2):357–64. 10.1158/1078-0432.CCR-14-1374 25388162 PMC4513664

[86] Xia Y Chang T Wang Y: YAP promotes ovarian cancer cell tumorigenesis and is indicative of a poor prognosis for ovarian cancer patients. *PLoS One.* 2014;9(3):e91770. 10.1371/journal.pone.0091770 24622501 PMC3951505

[87] Sun D Li X He Y: YAP1 enhances cell proliferation, migration, and invasion of gastric cancer *in vitro* and *in vivo*. *Oncotarget.* 2016;7(49):81062–76. 10.18632/oncotarget.13188 27835600 PMC5348376

[88] Wu C Xu B Yuan P: Genome-wide interrogation identifies *YAP1* variants associated with survival of small-cell lung cancer patients. *Cancer Res.* 2010;70(23):9721–9. 10.1158/0008-5472.CAN-10-1493 21118971

[89] Han S Wang D Tang G: Suppression of miR-16 promotes tumor growth and metastasis through reversely regulating YAP1 in human cholangiocarcinoma. *Oncotarget.* 2017;8(34):56635–56650. 10.18632/oncotarget.17832 28915618 PMC5593589

[90] Tang Y Weiss SJ: Snail/Slug-YAP/TAZ complexes cooperatively regulate mesenchymal stem cell function and bone formation. *Cell Cycle.* 2017;16(5):399–405. 10.1080/15384101.2017.1280643 28112996 PMC5351930

